# Spatial distribution and bioaccumulation of polychlorinated biphenyls (PCBs) and polybrominated diphenyl ethers (PBDEs) in snails (*Bellamya aeruginosa*) and sediments from Taihu Lake area, China

**DOI:** 10.1007/s11356-017-8467-x

**Published:** 2017-01-26

**Authors:** Ge Yin, Yihui Zhou, Anna Strid, Ziye Zheng, Anders Bignert, Taowu Ma, Ioannis Athanassiadis, Yanling Qiu

**Affiliations:** 1 Department of Environmental Science and Analytical Chemistry, Stockholm University, 11418 Stockholm, Sweden; 2 State Key Laboratory of Pollution Control and Resource Reuse, College of Environmental Science and Engineering, Tongji University, Shanghai, 200092 China; 3 Department of Chemistry, Umeå University, 90187 Umeå, Sweden; 4 Swedish Museum of Natural History, Box 50007, 10405 Stockholm, Sweden; 5 Key Laboratory of Yangtze River Water Environment (Ministry of Education), College of Environmental Science and Engineering, Tongji University, Shanghai, 200092 China; 6 College of Biology and Environmental Sciences, Jishou University, Jishou, 416000 China

**Keywords:** Persistent organic pollutants, Total organic carbon, Biota-sediment accumulation, Benthic organisms, Spatial distribution

## Abstract

Taihu Lake area is one of the densest metropolitan areas in the world including diverse industrial activity. In the present study, the snail (*Bellamya aeruginosa*) and sediment were collected from the Taihu Lake area to investigate the contamination status, congener pattern, spatial distribution, and bioaccumulation effect of polychlorinated biphenyls (PCBs) and polybrominated diphenyl ethers (PBDEs). The samples underwent liquid extraction, lipid removal by sulfuric acid, and acidic silica gel column, and subsequently analyzed by gas chromatography-electron capture detector (GC-ECD) and gas chromatography-mass spectrometry (GC-MS). Concentration of ∑_22_PCBs ranged between 90 and 680 ng g^−1^ lipid weight in the snails and between 0.018 and 0.82 ng g^−1^ dry weight in the sediments. Concentration of ∑_24_PBDEs varied from 25 to 200 ng g^−1^ lipid weight in the snails and from 0.62 to 67 ng g^−1^ dry weight in the sediments. The levels of PCBs and PBDEs observed were in the medium to low range compared with other studies in the world. CB-153 was the predominant PCB congener in both snails and sediments whereas BDE-209 showed a low bioavailability in the snails, even if it contributed up to 70% of ∑_24_PBDEs in the sediments. The spatial distribution showed that the highest concentration of PCBs and PBDEs were detected in samples from Zhushan Lake. East Taihu Lake and Dianshan Lake showed lower concentration of PCBs and PBDEs than the other sampling sites. Biota-sediment accumulation was found between snails and sediments of most of PCB and PBDE congeners except for the highly brominated BDEs (i.e., BDE-209). Therefore, sediment is suggested to be an appropriate matrix to monitor BDE-209 while aquatic species such as the snail could be good for monitoring of PCBs and lower brominated BDE congeners. No significant correlation (Spearman correlation test, two-tailed) of CB-153 (*r* = 0.54, *p* = 0.27) or BDE-47 (*r* = 0.60, *p* = 0.21) was found between snails and sediments.

**Electronic supplementary material:**

The online version of this article (doi:10.1007/s11356-017-8467-x) contains supplementary material, which is available to authorized users.

## Introduction

Persistent organic pollutants (POPs) are of major global concern due to their persistency, bioaccumulative properties, and their toxicity to humans and wildlife (UNEP 2016). To date, there are 26 POPs listed under the Stockholm Convention, divided into three categories, i.e., organochlorine pesticides (OCPs), industrial chemicals, and unintentional byproducts (UNEP 2015). Polychlorinated biphenyls (PCBs) and polybrominated diphenyl ethers (PBDEs) are two of the most well-known POPs from industrial production listed in the Stockholm Convention. The production of PCBs started in 1929 and due to their resistance to degradation and low flammability, they were widely used as, i.e., insulator oil in transformers and capacitors, as additives in sealants, and as heat transfer agents (Erickson and Kaley 2011; Hagmar 2003). The production of PBDEs started in the 1970s and they have since been widely used as additive flame retardants in, i.e., electronic equipment, textiles, and construction materials (Chen and Hale 2010; Sjodin et al. 2003). The PBDEs have been manufactured as three technical mixtures, i.e., penta-, octa- and deca-BDE (La Guardia et al. 2006). Penta- and octa-BDE are today listed and banned under the Stockholm Convention, while deca-BDE currently is under review to be included (UNEP 2015). Still, deca-BDE has been phased out in both Europe and North America but is still manufactured in China (Newton 2015). Because of their long historic usage and lipophilic properties, these POPs have been ubiquitously detected in environmental compartment such as sediment, soil, aquatic organisms, and human milk (Cai et al. 2008; Covaci et al. 2003; Sjodin et al. 2003; Ueno et al. 2010).

Sediments act both as a source and sink of POPs in the aquatic environment (Yang et al. 2014). When released into lakes via atmospheric deposition and surface runoff, POPs tend to adsorb to particles in the sediment (Nhan et al. 2001). Simultaneously, these POPs can be released from the sediment-water interface under certain conditions and then ingested by benthic organisms and further accumulate in the food chains (Zhao et al. 2009). Benthic invertebrates are considered as good indicator species for local environmental pollution since they have a wide geographic distribution and a low mobility (Goldberg et al. 1978; Ramu et al. 2007b). Mussels have been widely applied to investigate environmental contamination of POPs and heavy metal (Giandomenico et al. 2013; Ramu et al. 2007a; Yin et al. 2015). However, in the Taihu Lake area, mussels are commonly cultivated for pearl production and the natural mussels are rarely found. Snails have also been used to assess the environmental exposure level of exogenous contaminants and their toxic effects (Ducrot et al. 2014; Nhan et al. 2001; Senthilkumar et al. 2001). *Bellamya aeruginosa* is a freshwater benthic gastropod, and is commonly present in Chinese freshwater ecosystems. As a deposit-feeder, *B. aeruginosa* is closely associated to the surface sediments, where they burrow in the upper layer and feed on particulate matters (Chen and Song 1975). At present, this species has been proposed as a potential test species for sediment toxicity assessments (Ma et al. 2010). Still, in studies investigating the environmental exposure of contaminants, the snails have shown to play a minor role in terms of bioaccumulation and biomagnification in the aquatic food webs (Kobayashi et al. 2015; Senthilkumar et al. 2001; She et al. 2013; Zhu et al. 2015). Research concerning spatial distribution of POPs in snails and whether or not snail can be used as biomonitoring species is less abundant.

Taihu Lake is located in the Yangtze River Delta and is the second-largest freshwater lake in China with an area of 2250 km^2^ and an average depth of 2 m. The lake is surrounded by the cities of Changzhou, Wuxi, and Suzhou in the Jiangsu province, and the city of Huzhou in the Zhejiang province. Dianshan Lake, with an area of 62 km^2^, is situated downstream of Taihu Lake and is one of most important freshwater lakes in the Taihu Lake area since it is one of the drinking water reservoirs for the citizens of Shanghai (Zhou et al. 2016a). With the recent increase in industrialization and urbanization, the Taihu Lake area has suffered from excessive inputs of chemicals, including chemical manufacturing plants such as production of textile, pharmaceuticals, pesticides, flame retardants, and other fine chemicals. Historically, the northwestern part of Taihu Lake, i.e., Suzhou, Wuxi and Changzhou have suffered more from environmental pollution than the eastern part (Ma et al. 2013). So far, the efforts made to improve the pollution situation have focused on the eutrophication and water quality rather than on the contamination of POPs (Pan et al. 2011). In addition, in terms of POPs, OCPs have been taken priority for consideration because of the historic application (Zhao et al. 2009). Hence, it is necessary to look into industrial POPs such as PCBs and PBDEs.

The objectives of the present study were to assess the contamination status, congener pattern, and spatial distribution of industrial POPs, i.e., PCBs and PBDEs in the Taihu Lake area using snails (*B. aeruginosa*) and sediments. Biota-sediment accumulation factors (BSAF) were calculated to investigate the bioaccumulation potential of PCBs and PBDEs in the snails. In addition, the possibility of introducing the snail as an indicator species for environmental monitoring of POPs was studied.

## Material and methods

### Sampling

**Fig. 1:**
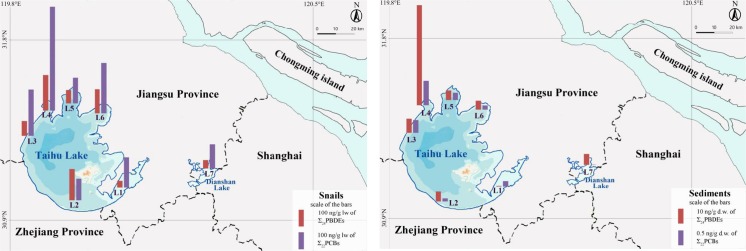
Spatial distribution of the sum of 22 congeners of polychlorinated biphenyls (∑_22_PCBs) and the sum of 24 congeners of polybrominated diphenyl ethers (∑_24_PBDEs) in snails (*Bellamya aeruginosa*) (*left*) and sediments (*right*) from the coastal area of Taihu Lake

Snail and sediment samples were collected from the Taihu Lake area, located in the Yangzte River Delta. The sampling sites were located in Taihu Lake (the eastern (L1), western (L2 and L3), and northern parts (L4–L6)) and in Dianshan Lake (L7) (Fig. 1). All sampling took place in May 2014 and detailed descriptions of the sampling sites are given in Table S1. One sediment pool and two snail pools (one female and one male) from each location were used for chemical analysis. The sediment pools were prepared from equal weights of five subsamples from each sampling site. Snails (*B. aeruginosa*) were sampled together with 10 L of lake water from the same locations as the sediments. The snails were immediately transported to the laboratory where an air pump was placed in the lake water for 24 h for gut purging. Males and females were separated by comparing their tentacles. The males have a vault-shaped, stubby right tentacle whereas females have a pair of uniform tentacles (Ma et al. 2010). Each pooled snail sample consisted of the soft tissue of five individual snails. All samples were stored at −20 °C prior to chemical analysis.

### Chemicals and standards

Seven PCB congeners (CB-28, -52, -101, -118, -138, -153, and -180) were purchased as a mixture from Larodan Fine Chemicals (Malmö, Sweden). A PCB mixture containing octa-CBs (CB-194, -195, -196, -197, -198, -199, -201, -202, -203, -204, and -205); nona-CBs (CB-206, -207, and -208); and CB-209 were purchased from AccuStandard (New Haven, USA). BDE-28, -47, -66, -99, -100, -153, -154, and -183 were bought as a mixture from Wellington Laboratories Inc. (Guelph, Ontario, Canada). A mixture with octa- to deca-BDEs (BDE-194, -195, -196, -197, -198, -199, -200, -201, -202, -203, -204, -205, -206, -207, -208, and -209) were also bought from Wellington Laboratories Inc. (Guelph, Ontario, Canada). CB-200 from AccuStandard (New Haven, USA) and BDE-139 from Wellington Laboratories Inc. (Guelph, Ontario, Canada) were used as surrogate standards. 1,1-dichloro-2,2-diphenylethene (*ϕ*-DDE) synthesized in house and BDE-138 from Wellington Laboratories Inc. (Guelph, Ontario, Canada) were used as volumetric standards. All solvents, acids, and salts used were of highest quality commercially available. Silica gel (0.063–0.2 mm) purchased from Merck (Darmstadt, Germany) was activated at 300 °C overnight prior to use.

### Extraction and clean-up

Homogenized snail intestine (3–5 g) and sediments (15–20 g) were spiked with surrogate standards CB-200 (5.2 ng) and BDE-139 (3.4 ng) prior to extraction. Snails were extracted using a solid-liquid extraction following procedures described elsewhere with minor modification that *n*-hexane was replaced with *iso*-hexane as solvent (Jensen et al. 2009). The lipids were determined gravimetrically after evaporation. For the sediments, extraction and sulfur removal was done as described elsewhere (Nylund et al. 1992). Lipids and organic matters were removed using concentrated sulfuric acid (98%), and further clean-up was carried out using a Pasteur pipette packed with activated silica gel (0.1 g) and activated silica (0.9 g) impregnated with concentrated sulfuric acid (2:1 *w*/*w*) on top. The columns were conditioned with *n*-hexane (3 mL), the extract was added, and the analytes were eluted with *n*-hexane/dichloromethane (15 mL, 1:1, *v*/*v*). The volume was reduced and the solvent was changed to *n*-hexane (final volume 0.2 mL) by a gentle stream of nitrogen. BDE-138 (4 ng) and *ϕ*-DDE (4 ng) was added as a volumetric standard prior to gas chromatography-mass spectrometry (GC-MS) and GC-electron capture detector (ECD) analysis, respectively.

### Instrumental analysis

PCBs (CB-28, -52, -101, -118, -138, -153, and -180) were analyzed using a Varian 450 gas chromatograph equipped with an electron capture detector (GC-ECD) and a Varian CP-8400 auto-sampler. Injections (1 μL) were done on a programmable temperature vaporizing (PTV) injector operating in splitless mode at a temperature of 250 °C. The column used was a BP5 (30 m × 0.25 mm i.d. × 0.25 μm film thickness; SGE Analytical Science) with helium as a carrier gas and nitrogen as make-up gas. The column oven temperature program was 80 °C for 2 min, 15 °C/min, to 300 °C (18 min).

PBDEs and highly chlorinated PCBs (octa- to deca-PCBs) were analyzed using a Varian-450 gas chromatograph coupled to a Varian 320 mass spectrometer (GC-MS) using electron capture negative ionization (ECNI) and selective ion monitoring (SIM). Bromine ions (*m*/*z* 79 and 81) were scanned for quantification of PBDEs. For the highly chlorinated PCBs, molecular ions (427.7:429.7 for octa-CBs, 461.6:463.6 for nona-CBs, and 497.7:499.7 for deca-CB) were selected for identification and quantification. The GC was equipped with a CTC GC Pal auto-sampler and a Varian 1079 PTV injector. Injections (1 μL) were done on a DB-5HT (30 m × 0.25 mm i.d. ×0.10 μm film thickness; Agilent J&W) GC column, with methane (scientific 5.5, AGA Stockholm, Sweden) as reagent gas. The PTV injector was operated in splitless mode at a temperature of 260 °C. Helium was used as carrier gas at a constant flow of 1.0 mL/min. The oven program was 55 °C for 2 min, 15 °C/min, to 320 °C and hold for 4 min. The ion source and transfer line temperature were set at 230 and 300 °C for PBDEs whereas 180 and 280 °C for highly chlorinated PCBs, respectively.

### TOC measurement and BSAF

Total organic carbon (TOC) corrected data in sediment have been reported to correspond to the concentration on lipid weight basis in organisms (Bierman 1990). Approximately 10 mg of freeze-dried sediment samples were treated with acid and combusted with a Carlo Erba NC 2500 analyzer. The relative error was below 1% for %C and %N measurements.

Biota-sediment accumulation factors (BSAF) (Burkhard 2009) were calculated from three measured variables (Eq. 1):

1 $$ \mathrm{BSAF}={C}_o/\left({C}_s/\mathrm{TOC}\right) $$

*C*_*o*_ is the concentration in the organism (ng g^−1^ lipid weight (lw)), *C*_*s*_ is the concentration in sediment (ng g^−1^ dry weight (dw)), and TOC is defined as the fraction of organic carbon (%) in the sediment. A theoretical BSAF value between 1 and 2 has been suggested to be used as a threshold value for bioaccumulation (Burkhard 2009), and in the present study, BSAF = 2 was chosen as the limit value.

### Quality control and quality assurance

One solvent blank sample was analyzed in parallel with each batch of six samples to keep track of any potential contamination. Two Baltic Sea herrings were analyzed together with the samples as a working laboratory standard, and the results of HCB, CB-138, and CB-153 were within the region of acceptance (mean value ± standard deviation) in a control chart. The limit of detection (LOD) was set to three times the background noise (*S*/*N* = 3) and the limit of quantification (LOQ) was set to 10 times (*S*/*N* = 10) the background noise or three times the average value of the blank samples. Small amounts of BDE-47 were detected in the blanks and were subtracted from the samples. The recoveries (mean **±** standard deviation) of the surrogate standards were 78 **±** 10% for CB-200 and 89 **±** 7% for BDE-139, respectively. For the GC-MS (SIM) analysis of highly chlorinated PCBs, isotopic ratios of two quantitative ions for each compound had to be within 15% of the theoretical chlorine value. All identification and quantification were based on comparisons to authentic reference standards.

### Statistical analysis

Statistical tests were conducted using SPSS 22.0. Samples below the established LODs were considered as zero, while samples between the LOD and LOQ were set to half of the LOQ value in statistical calculations. Paired *t* test was used to examine any sexual differences of PCB and PBDE congeners in snails. To assess any correlations between PCB and PBDE congeners in sediments, the Spearman correlation test (two-tailed) was used. The significant level was set to *α* = 0.05 for all tests. Principal component analysis (PCA) was carried out to compare the PBDE congener pattern to technical PBDE products. The original data was centralized and normalized to remove differences based on levels. The PCA was firstly conducted on six PBDE commercial products (La Guardia et al. 2006). Subsequently, the snail and sediment samples were projected on the PCA bi-plot.

## Results

Concentrations (mean and range, min–max) of PCBs and PBDEs in snails and sediments are presented in Table 1 on a lipid weight basis (ng g^−1^ lw) and on dry weight basis (ng g^−1^ dw). Individual concentrations in snails and sediments are given in Table S2 and S3, respectively. Concentrations of ∑_22_PCBs in snails ranged from 90 to 680 ng g^−1^ lw (mean value 250 ng g^−1^ lw). The congener profile of PCBs is shown in Fig. S1. CB-153 was the most abundant congener, accounting for 13 to 54% (average 33%) of ∑_22_PCBs. CB-138, -118, and -101 were other major congeners in the snails, accounting for 20, 11, and 9.0% on average to ∑_22_PCBs, respectively. CB-28 was not detected in any of the snail samples. Among the higher chlorinated congeners (octa- to deca-CBs) analyzed CB-209 and CB-196/-203 were the major ones in snails accounting for 4.5 and 2.7% of ∑_22_PCBs, respectively.

**Table 1:** A global comparison of concentrations (mean and range in ng g^−1^ lw for snail and ng g^−1^ dw for sediment) of polychlorinated biphenyls (PCBs) and polybrominated diphenyl ethers (PBDEs) in snails and sediments

Location	Sampling year (*n*)	Species	Unit	PCBs				PBDEs				
			ng g^−1^	CB-138	CB-153	CB-209	∑_7_PCBs^a^	BDE-47	BDE-99	BDE-153	BDE-209	∑_8_PBDEs^b^
Snail												
Taihu Lake area	2014 (14)	*Bellamya aeruginosa*	lw	54 (15–180)	72 (18–130)	11 (2.1–40)	180 (79–590)	13 (4.9–24)	13 (4.1–22)	8.1 (1.9–16)	20 (ND–57)	49 (19–84)
Ariake Sea, Japan	2012 (1)	*Certithidea rhizophorarum*	lw	130	ND	74	580	6.8	2.2	6.8	74	31
South China	2010 (12)	*Ampullariidae* apple snail	lw					14 (2.7–34)	16 (4.6–38)	4.6 (0.82–17)	61 (41–150)	52 (41–270)
Baiyangdian Lake, China	2007 (2)	*Viviparus* river snail	lw					0.70	0.83	0.35		8.8
South India	1998 (25)	*Augur territella* cone snail	lw				2900^c^					
South India	1998 (11)	*Ampullariidae* apple snail	lw				660^c^					
Arctic Lakes, USA	1993 (7)	Snail (*Lymnea* sp.)	lw	67	50	ND	350					
Hanoi, Viet Nam	1997 (8)	*Angulyagra* sp.	lw	210 (70–570)	220 (76–700)		740 (210–2100)					
Sediment												
Taihu Lake area	2014 (7)		dw	0.029 (0.013–0.076)	0.079 (0.045–0.18)	0.044 (0.007–0.19)	0.23 (0.097–0.57)	0.019 (0.009–0.033)	0.045 (ND–0.087)	0.024 (ND–0.078)	13 (0.048–56)	0.28 (0.067–0.92)
Hanoi, Viet Nam	1997 (12)		dw	1.7 (0.15–4.3)	2.1 (0.27–4.8)		7.7 (0.61–21)					
Taihu Lake	2002 (10)		dw	0.082 (0.050–0.14)	0.090 (0.047–0.18)	0.059 (0.033–0.11)	0.58 (0.32–1.1)					
Almeria, Spain	2004 (4)		dw					0.095 (0.07–0.12)	0.10 (0.05–0.14)	ND	3.1 (2.5–4.0)	0.42 (0.12–0.59)
Barcelona, Spain	2004 (4)		dw					0.098 (0.07–0.13)	0.17 (0.14–0.22)	0.26 (0.24–0.32)	58 (3.0–130)	1.4 (0.95–2.1)
Netherlands	2003 (22)		dw					1.1 (0.30–7.1)	0.60 (0.20–5.5)	0.70 (0.10–5.0)	22 (4.0–510)	
Beijiang River, China	2006 (40)		dw					0.044 (0.005–71)	0.03 (0.003–45)	0.01 (ND–15)	5.2 (0.23–1600)	0.11 (0.019–180)
Chicago sanitary and ship canal, USA	2013 (10)		dw	29 (1.6–67)	22 (1.2–41)	1.1 (0.11–2.1)	350 (15–710)	15 (2.0–25)	16 (0.79–35)	1.7 (0.10–4.1)	83 (2.2–210)	38 (2.9–68)
East Lake, Wuhan, China	2013 (126)		dw	ND	ND		18 (ND–110)	11 (ND–24)	12 (ND–41)	ND		45 (9.7–83)
Scheldt estuary	2011 (36)		dw	0.43 (0.08–34)	0.50 (0.07–36)	0.03 (0.03–0.83)	1.9 (0.47–140)	0.10 (0.03–1.3)	0.04 (0.03–1.6)	0.01 (0.01–0.21)	6.8 (0.43–1200)	0.18 (0.10–2.8)
Chaohu Lake, China	2009 (49)		dw	0.21 (ND–0.83)	0.05 (ND–0.20)			0.31 (0.16–0.57)	0.32 (ND–0.77)	0.03 (ND–0.35)	6.5 (0.31–75)	0.84 (0.16–2.7)
Pearl River Delta, China	2013 (38)		dw	2.5 (ND–15)	3.3 (ND–15)		48 (9.9–150)					

*ND* not detected, *lw* lipid weight, *dw* dry weight

^a^Sum of CB-28, -52, -101, -118, -138, -153, and -180

^b^Sum of BDE-28, -47, -66, -99, -100, -153, -154, and -183

^c^PCBs congeners are not stated in the paper

Concentrations of ∑_24_PBDE in the snails ranged from 25 to 200 ng g^−1^ lw (mean value 110 ng g^−1^ lw) and were lower than ∑_22_PCBs in all samples. The congener profile of PBDEs is shown in Fig. S2. BDE-47 and BDE-99 were two of the main congeners in the snail samples, accounting for 14 and 13% of ∑_24_PBDE, respectively. BDE-209 was detected in 10 out of 14 snail samples. No significant difference (*p* > 0.05) was found between male and female snails in Taihu Lake for PCB and PBDE congeners analyzed.

Concentration of ∑_22_PCBs in the sediments ranged from 0.018 to 0.82 ng g^−1^ dw (mean value 0.26 ng g ^−1^ dw). The congener profile of PCBs for snails and sediments was quite similar, with CB-153, -138, and -101 as the major congeners (Fig. S1).CB-209 was quantified in all sediment samples analyzed and accounted for 4.5% to ∑_22_PCBs.

Concentration of ∑_24_PBDEs in the sediments ranged from 0.63 to 67 ng g^−1^ dw (mean value 15 ng g ^−1^ dw). BDE-209 was the major PBDE congener in the sediments, followed by BDE-207 and BDE-206. Concentration of BDE-209 in sediments ranged from 0.45 to 56 ng g^−1^ dw, with a mean concentration of 12 ng g^−1^ dw. No correlation (Spearman correlation test, two-tailed) was observed between the TOC and ∑_22_PCBs (*r* = 0.71, *p* = 0.11) or between the TOC and ∑_24_PBDEs (*r* = 0.77, *p* = 0.072).

## Discussion

### Congener profile

The highly chlorinated PCBs were abundant in both snails and sediments analyzed in the present study, accounting for 15 and 13% of ∑_22_PCBs in snail and sediment samples, respectively. CB-209 has previously been reported with a 100% detection frequency in apple snail (*Ampullariidae*) from an electronic waste recycling site in South China (Fu et al. 2011). Highly chlorinated PCBs have also been reported in bird eggs, snake, eel, and frog (Zhou et al. 2016b, c), indicating the ubiquitous contamination pattern in wildlife in Yangtze River Delta. The source of the highly chlorinated PCBs might be from the use of technical products similar to Aroclor 1268 (Kannan et al. 1997) and/or from any other non-Aroclor sources, i.e., from formation in the manufacture of phthalocyanine green pigments used in paints (Hu and Hornbuckle 2010) or in the process of titanium dioxide purification (Rowe et al. 2007). Shang et al. (2014) analyzed azo-type pigment from Chinese market and found CB-11 was the dominant PCB congener whereas CB-209 was only detected in trace amounts. In addition, highly chlorinated PCBs can be produced as a byproduct from combustion processes (Liljelind et al. 2003).

In both snail and sediment samples, CB-153, and -138 were the primary PCB congeners (Fig. S1), similar to other studies on, i.e., sediment (len-Gil et al. 1997), Greenland shark (len-Gil et al. 1997; Strid et al. 2007), human serum (Linderholm et al. 2010). These congeners share the structural characteristics that no adjacent *meta-/para-*position are entirely substituted by hydrogen, and thus more resistant to metabolism by cytochrome P450-dependent monooxygenase system (Sundstrom et al. 1976).

For PBDEs, BDE-47 and BDE-99 were the major congeners (c.f. Table S2) in snails, followed by BDE-100. This is a typical PBDE congener profile often observed in aquatic species most likely due to the historic usage of the commercial Penta-BDE product (de Boer et al. 2003; Strid et al. 2010; Yin et al. 2015). The average concentration ratio between BDE-99 and BDE-100 in the snails was 2.2, slightly lower than the ratio in technical Penta-BDE mixtures (3.7 and 5.7 in DE-71 and Bromkal 70-5DE, respectively) (La Guardia et al. 2006). It could be due to the possible higher biodegradability of BDE-99 compared to BDE-100 (Mizukawa et al. 2013). In common carp exposed to commercial Penta-BDE mixture via their food, it was found that BDE-99 degraded to BDE-47 whereas BDE-100 was resistant to debromination, indicating that structure-selective debromination was occurring (Zeng et al. 2012).

BDE-209 was the predominant PBDE congener in the sediments, accounting for 72–84% of ∑_24_PBDEs, followed by the nona-BDE congeners (i.e., BDE-207 and BDE-206). This is in accordance with other studies where BDE-209 was also the predominant PBDE congener in sediment (de Boer et al. 2003; Fu et al. 2011). Apart from highly brominated PBDEs, BDE-183 was also abundant in the sediments. BDE-183 is one of the main congeners in Octa-BDE technical products (i.e., in Bromkal 79-8DE) (La Guardia et al. 2006).

**Fig. 2:**
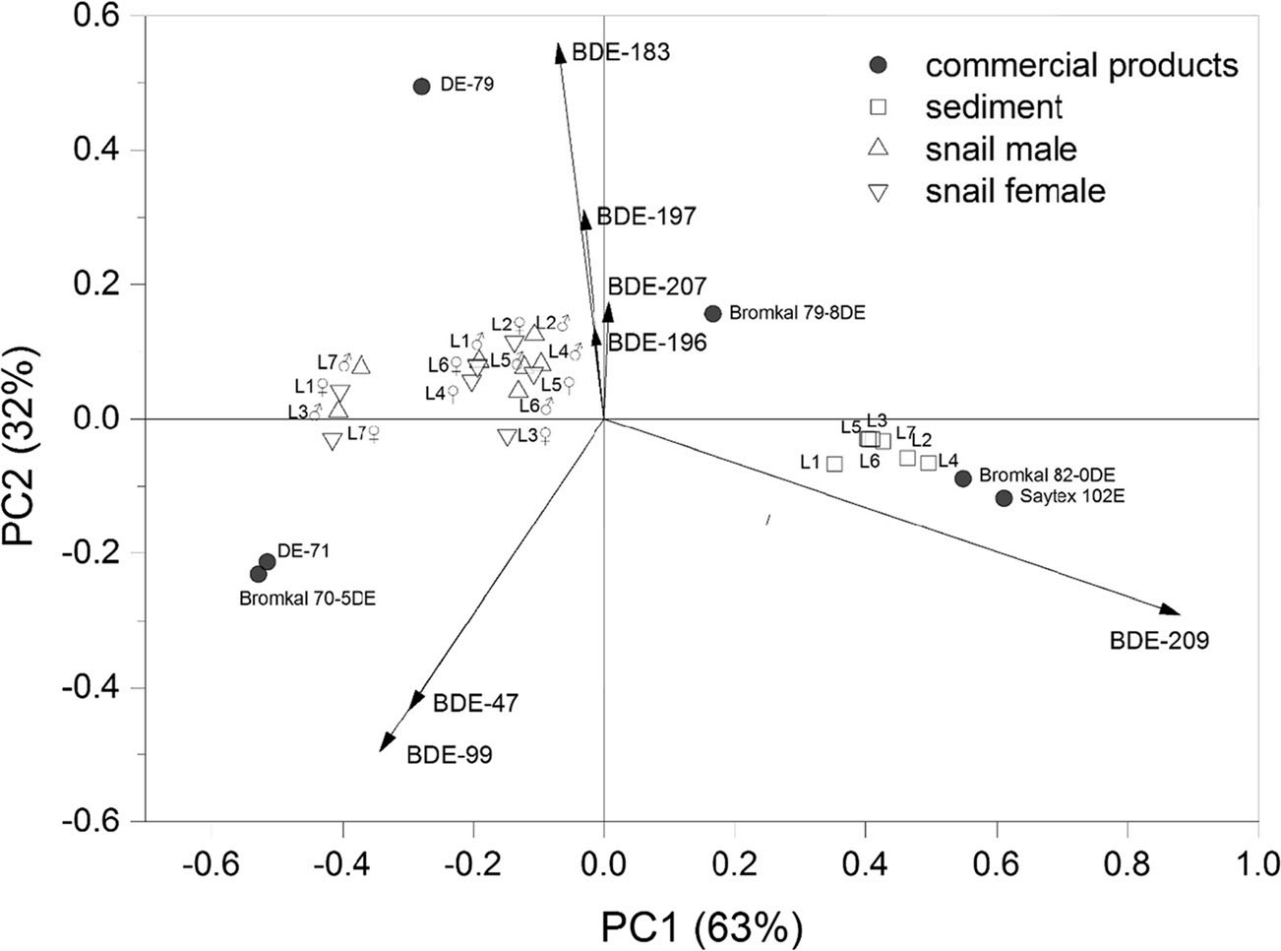
Principal component analysis (PCA), comparison of the congener profile of polybrominated diphenyl ethers (PBDEs) in snails (*Bellamya aeruginosa*) and sediment to commercial technical mixtures of PBDEs (DE-71 and Bromkal 70-5DE are Penta-BDE; DE-79 and Bromkal 79-8DE are Octa-BDE; and Saytex 102E and Bromkal 82-0DE are Deca-BDE commercial product)

The comparison between commercial PBDE products with snails and sediments by PCA were shown in Fig. 2. The first two principal components (PC) explained 95% of the variance in the data (63% for PC1 and 32% for PC2, respectively). The factor loadings were presented in Table S4. For PC1, the loading vector of BDE-209 was positive (0.88), which lead to deca-BDE distributed in the positive direction in the *x*-axis. For PC2, BDE-183 and BDE-197 have positive value whereas BDE-47 and BDE-99 have negative value. This results in the octa-BDE located in positive direction in the *y*-axis whereas Penta-BDE located in the negative direction in the *y*-axis (Fig. 2). The bi-plot of score and vector loading indicate that the sediment samples were grouped together close to deca-BDE and one of the octa-BDE (Bromkal 79-8DE) mixtures regardless of the sampling sites. The snail samples on the other hand, were projected between the penta- and octa-BDE mixtures (Fig. 2). The differences seen regarding patterns of PBDEs in snail and sediment samples may be explained by a lower bioavailability of the highly brominated PBDEs, in particular for BDE-209, in the snails. Similar PBDE congener differences were observed among biota samples, suspended particulate matter, and sediment in the Netherlands (de Boer et al. 2003), in which BDE-47 and BDE-99 were detected as primary congeners in mussels whereas BDE-209 was predominant in the other two compartments. In short, the ratio of major congeners together with PCA results implies future study for source identification needs to be assessed.

Spearman rank correlation coefficients for selected PCB and PBDE congeners in sediments are given in Table S5. CB-101, -138, and -153 were not correlated either to PBDEs, or to each other (*p* ˃ 0.05). BDE-47 was strongly correlated to BDE-99 (*r* = 0.94, *p* = 0.005) and BDE-100 (*r* = 0.89, *p* = 0.019), indicating these congeners might be from the same source, i.e., from previous use of penta-BDE. It is worthy to note that CB-209 correlated to the higher BDE congeners (*r* > 0.80, *p* < 0.05). As mentioned above, one of the potential sources of CB-209 is from Aroclor mixtures with high chlorine content (i.e., Aroclor 1268). These products might have been used as flame retardants due to low flammability and low chemical reactivity (Wright and Beacham 1969). In addition, because of the high chlorine content, the physical-chemical properties (i.e., molecular weight, log *K*_ow_) of CB-209 resemble more to PBDEs than other PCB congeners. CB-209 was previously detected in an e-waste site from China, indicating the application of highly chlorinated PCBs with a similar function as PBDEs in electronic equipment (Wu et al. 2008).

### Spatial distribution

The spatial distribution of ∑_22_PCBs together with ∑_24_PBDEs in snails and sediments are shown in Fig. 1, respectively. It is apparent that Zhushan Lake (L4) showed the highest concentration of PCBs (0.82 ng g^−1^ dw in sediment and 610 ng g^−1^ lw in snail) and PBDEs (67 ng g^−1^ dw in sediment and 210 ng g^−1^ lw in snail). L4 has been considered as one of the most eutrophicated sites in Taihu Lake (He et al. 2012). In addition, other studies have shown that levels of polycyclic aromatic hydrocarbons (He et al. 2013) and mercury (Hu et al. 2014) is the highest in L4 compared to other locations investigated in the northern Taihu Lake. This implies that the contamination status in this part of the lake is highly influenced by anthropogenic activities.

The lowest concentrations of PCBs and PBDEs were present in the eastern Taihu Lake (L1). L1 is located close to the main outlet from Taihu Lake, and it is possible that anthropogenic pollutants are further transported into the canal system rather than accumulating in this area. In addition, He et al. measured the nitrogen isotope ratios (*δ* ^15^N) in *B. aeruginosa* from four sites of Taihu Lake (He et al. 2012). They observed that δ^15^N level in L1 is lower relative to those levels in L4–L6 and this result could be explained by different food sources (He et al. 2012). *B. aeruginosa* feed on not only fouling organisms and organic detritus but also phytoplantkton. East Taihu Lake (L1) is a grass-type ecosystem and thus, the snails mainly feed on aquatic plant debris whereas in the north and western parts of Taihu Lake (L2–L6) the snails mainly feed on algal organic matter in the sediment surface (He et al. 2012).

For the other four sampling sites in Taihu Lake, Xiaomeikou (L2), Dapukou (L3), Meiliang Bay (L5), and Gong Lake (L6), levels of PCBs and PBDEs were quite evenly distributed. L2 and L3 are located in the western parts of Taihu Lake, close to the cities of Huzhou and Yixing (affiliated to Wuxi), respectively. More chemical industries are located in the western parts of Taihu Lake than in the eastern parts. Further, there are also several rivers running into the lake in the western parts, possibly bringing contaminants into the lake. L5 and L6, located in the northern part of the Taihu Lake, are close to the city of Wuxi, a city with a high urbanization rate and population density. Historically, Wuxi city was heavily contaminated by anthropogenic pollutants (Liu et al. 2009; Lu et al. 2013), but the situation has improved since the local governments made efforts to a more effective sewage water treatment. Today, the city has 6.5 million inhabitants and L5 is in fact one of its major drinking water sources.

Dianshan Lake (L7) showed low concentrations of both PCBs and PBDEs in snails, similar to the snails from L1, indicating less industrial activity in this area. Dianshan Lake is one important source for drinking water to the inhabitants of Shanghai, and further, also an important lake for protection and ecological conservation (Wang et al. 2013). The spatial distribution pattern implies that the outlet and downstream of Taihu Lake suffered less contamination from industrial POPs than other parts of Taihu Lake.

### Comparison with other studies

A global comparison of PCB and PBDE concentrations in snail and sediment samples are given in Table 1. For PCBs, the sum of the seven congeners commonly reported in the literature (∑_7_PCBs: CB-28, -52, -101, -118, -138, -153, and -180) are presented together with concentrations of CB-138, CB-153, and CB-209, if available. Similarly are the sum of eight PBDE congeners (∑_8_PBDEs: BDE-28, -47, -66, -99, -100, -153, -154, and -183) presented together with concentrations of BDE-47, -99, -153, and BDE-209. In general, ∑_7_PCBs levels detected in the present study were in the lower end compared to the other studies included in Table 1. ∑_7_PCBs in snails in the present study ranged between 79 and 590 ng g^−1^ lw, with an average concentration of 180 ng g^−1^ lw, much lower than in snails from South India (*Augur territella*, 2900 ng g^−1^ lw) (Senthilkumar et al. 2001) and slightly lower than in snails from Japan (*Certithidea rhizophorarum*, 580 ng g^−1^ lw) (Kobayashi et al. 2015) and the USA (*Lymnea* sp., 350 ng g^−1^ lw) (len-Gil et al. 1997). This is in accordance with our previous finding in shellfish, showing that PCBs in shellfish (*Mytilus edulis*) in Yangtze River Delta is lower than in other parts of the world (Yin et al. 2015). ∑_7_PCBs in sediments (0.097–0.57 ng g^−1^ dw) in the present study were lower than in sediments from the Hanoi region, Vietnam (7.7 ng g^−1^ dw) (Nhan et al. 2001), the Chicago sanitary and shop canal in USA (35 ng g^−1^ dw) (Peverly et al. 2015), and national data from the Pearl River Delta, China (48 ng g^−1^ dw) (Lai et al. 2015). Levels of CB-209 in the present study is in accordance with what has been reported previously in Taihu Lake (Zhang and Jiang 2005).

∑_8_PBDE concentrations in the present study were in the moderate level compared to the other studies included in Table 1. The average concentration of ∑_8_PBDE in snails in the present study was 49 ng g^−1^ lw, with a range of 19–84 ng g^−1^ lw. This is similar to levels reported in snails from Japan (*Certithidea rhizophorarum*, 31 ng g^−1^ lw) (Kobayashi et al. 2015), and South China (*Ampullariidae*, 52 ng g^−1^ lw) (She et al. 2013). ∑_8_PBDE concentrations in sediments (0.067–0.92 ng g^−1^ dw) in the present study were slightly greater than those levels reported from Almeria, Spain (0.42 ng g^−1^ dw) (Eljarrat et al. 2005); Scheldt estuary (0.18 ng g^−1^ dw) (Van Ael et al. 2014); and Beijiang River, China (0.11 ng g^−1^ dw) (Chen et al. 2009), but lower than levels reported from hot spot areas such as the Chicago sanitary and shop canal in the USA (38 ng g^−1^ dw) (Peverly et al. 2015); the East Lake, China (45 ng g^−1^ dw) (Yun et al. 2015); and an e-waste dismantling site in Taizhou, China (8.2 ng g^−1^ dw) (Fu et al. 2011). BDE-209 concentrations in sediments ranged from 0.048 to 56 ng g^−1^ dw (mean 13 ng g^−1^ dw) in the present study and were comparable to Beijiang River, China (5.2 ng g^−1^ dw) (Chen et al. 2009) and the Chaohu Lake, China (6.5 ng g^−1^ dw) (Wang et al. 2012), but lower than Chicago sanitary and shop canal in USA (83 ng g^−1^ dw) (Peverly et al. 2015) and an e-waste dismantling site in Taizhou, China (460 ng g^−1^ dw) (Fu et al. 2011).

### Bioaccumulation

**Fig. 3:**
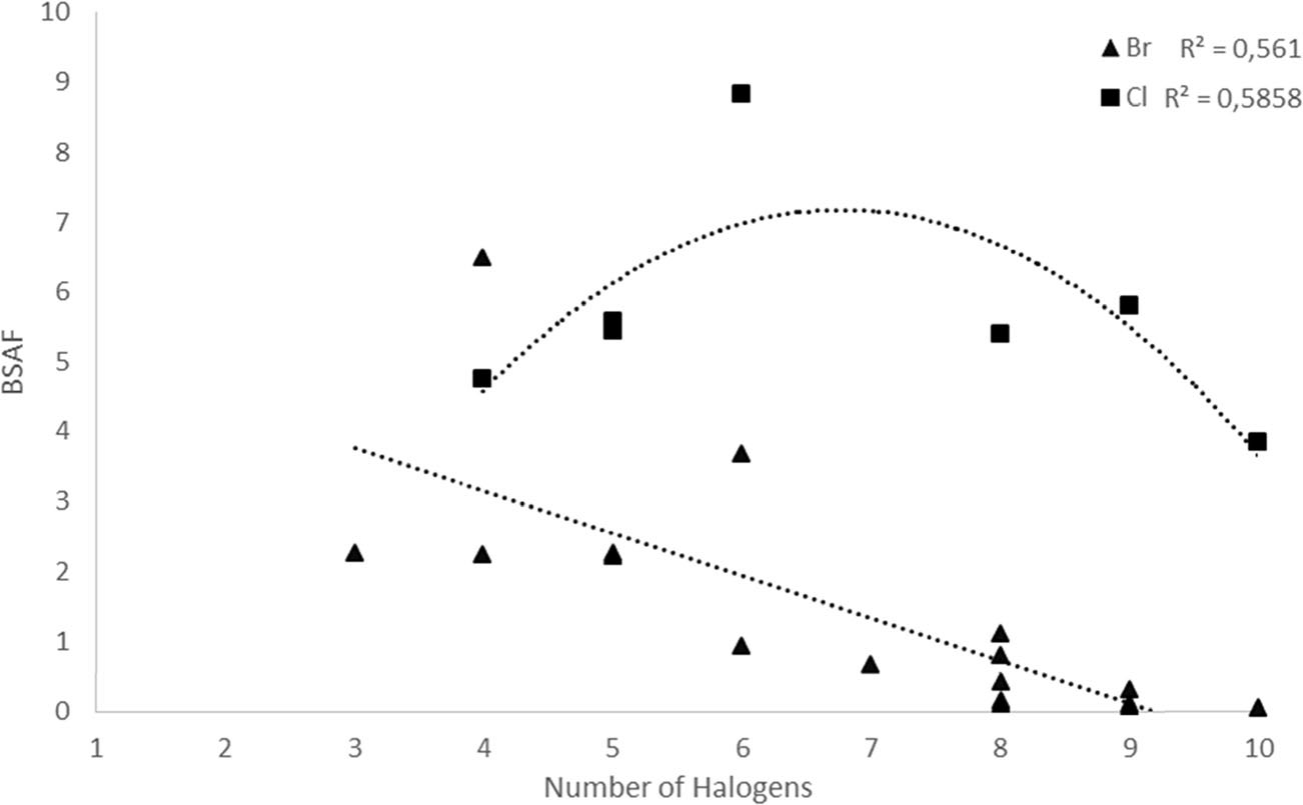
Regression relationship between biota-sediment bioaccumulation factors (BSAF) and the number of halogen atoms

Among the selected PCB and PBDE congeners, all of the PCB congeners and PBDE congeners with less than six bromines showed bioaccumulation potential whereas values below two were found for hexa- to deca-BDEs. The negative correlation between BSAF values and number bromine atoms for PBDEs is shown in Fig. 3 and Table S6. For PCBs, a parabola trend was observed in relation with BSAF and number of chlorines. It can be attributed to the higher metabolism and excretion potential for lower PCB congeners together with the reduced bioavailability for the higher PCB congeners (Yu et al. 2012). Such curvilinear phenomenon was in consistence with other studies. She et al. (2013) examined halogenated flame retardants in a herbivorous food chain (paddy soil-rice plant-apple snail) and found that the bioaccumulation factors between apple snail and rice plant increased for log *K*_ow_ values up to 7 or 8, and then started to decrease, which is likely due to their reduced bioavailability. Zhu et al. (2015) also found that bioaccumulation factors of PCBs in aquatic species start to decline from hepta-CBs corresponding to log *K*_ow_ values of 7 to 8. In the present study, higher BSAF values were in general found for PCBs compared to the PBDEs, implying that the PCBs have a higher bioavailability than PBDEs in the snails. This is, however, in contrast to a previous study where no bioaccumulation (BSAFs below one) occurred between snail (*B. aeruginosa*) and sediment for organochlorine pesticides (Zhao et al. 2009). There might be several reasons for the differences seen, i.e., the age of the snails since smaller (younger) snails may not have had sufficient time to accumulate contaminants to the same extent as older snails.

To compare the concentration of PCBs and PBDEs between snail and sediment, the concentration in sediment was normalized to organic carbon basis since the hydrophobic contaminants adsorbs to the organic carbon in sediment (Karickhoff 1981). Concentrations of ∑_24_PBDEs were detected at lower levels than that of ∑_22_PCBs in snail samples, whereas it shows the opposite way in sediment samples. In general, substances with higher *K*_ow_ value (e.g., PCBs) are likely to be adsorbed on organic matter in soil and sediment, and can migrate to fat tissues of living organisms in the aquatic environment. However, compounds with log *K*_ow_ values above 7 or 8, e.g., highly brominated PBDEs are often so strongly bound to the sediments preventing accumulation in the living organisms (She et al. 2013).

### Considering snail as a biomonitoring species

No correlations (Spearman correlation test, two-tailed) of the major congeners, i.e., CB-153 (*r* = 0.54, *p* = 0.27) and BDE-47 (*r* = 0.60, *p* = 0.21) were observed between snails and sediments. Sediments seem to be a good indicator for contaminants with larger molecular size (i.e., BDE-209) while snails could be used for biomonitoring of the lower brominated PBDE congeners. There are a number of factors supporting using snail for biomonitoring purposes. First of all, they are geographically widely distributed, easy to collect, and the movement during their lifespan is narrow. As a consequence, it is good to use snail for both temporal and spatial monitoring locally. In addition, they do not metabolize PCBs and PBDEs as much as species in high trophic levels (Nhan et al. 2001). Their position in the aquatic food web is also of importance since the snails are part of the diet for the black carp (*Mylopharyngodon piceus*), one of four favorable domestic fish species in China. In addition to this is the snails in Taihu Lake commonly consumed directly by humans. However, some other factors need to be taken into account. Snails have been suggested to be sensitive to exogenous pollutants. Oehlmann et al. (2000) exposed snail (*Marisa cornuarietis*) to bisphenol A, which is an endocrine disruptor compound at 1 ng/mL for 12 months and found bisphenol A induced a complex syndrome of alterations. As the industrial POPs level was much lower than the lethal concentration, snail may be used for long-term monitoring of POPs. Other influential factors, i.e., lipid content, species-specific difference, and age-related exposure levels should be further assessed.

## Conclusions

The present study showed moderate to low levels of PCBs and PBDEs in snail and sediment samples from the Taihu Lake area in China. CB-153, -138, and -101 were the major PCB congeners in both snails and sediment. BDE-47 was the main congener in snail whereas BDE-209 was the major PBDE congener in sediment. CB-209 was detected in all sediment samples and was significantly correlated to the highly brominated PBDEs. The spatial distribution showed that the contaminant load of PCBs and PBDEs in the northwestern part were higher compared to the eastern part of the Taihu Lake area (including the Dianshan Lake). Biota-sediment accumulation was found between snails and sediments for PCBs and for the lower PBDE congeners, but not for the highly brominated PBDEs. Therefore, sediment is suggested to be a good matrix to monitor BDE-209 while aquatic species such as the snail could be good for monitoring of lower brominated BDE congeners. Further research is needed to be able to assess the feasibility to use snails (i.e., *B. aeruginosa*) for environmental monitoring purposes.

## Electronic supplementary material

ESM 1 (DOCX 88.5 kb)
