# Supplementary material for: Spatial distribution and bioaccumulation of polychlorinated biphenyls (PCBs) and polybrominated diphenyl ethers (PBDEs) in snails (Bellamya aeruginosa) and sediments from Taihu Lake area, China
Source: Environ Sci Pollut Res Int. 2017 Jan 26;24(8):7740–51. doi: 10.1007/s11356-017-8467-x (PMC5383689; doi:10.1007/s11356-017-8467-x)
Supplement: Supplementary file 1 — (DOCX 88.5 kb) [file 11356_2017_8467_MOESM1_ESM.docx]

**Supplementary Material**

Spatial distribution and Bioaccumulation of Polychlorinated biphenyls (PCBs) and Polybrominated diphenyl ethers (PBDEs) in snails (*Bellamya aeruginosa*) and sediments from Taihu lake, China

Ge Yin^1^, Yihui Zhou^1,2^*, Anna Strid^1^, Ziye Zheng^1,3^, Anders Bignert^4,5^, Taowu Ma^6^, Ioannis Athanassiadis^1^, Yanling Qiu^5^

Corresponding author: [capitalzyh@yahoo.com](mailto:capitalzyh@yahoo.com)

Table S1. Detailed information and description of the sampling sites.

Table S2. Lipid content (%) and concentrations (ng g^-1^ lipid weight (lw)) of polychlorinated biphenyls (PCBs) and polybrominated diphenyl ethers (PBDEs) in snails (*Bellamya aeruginosa,* ♂: male and ♀: female) from Taihu lake area (Taihu lake (L1-L6) and Dianshan lake (L7)).

Table S3. Water content (%), total organic carbon (%) and concentration (ng g^-1^ dry weight (dw)) of polychlorinated biphenyls (PCBs) and polybrominated diphenyl ethers (PBDEs) in sediments from Taihu lake area (Taihu lake (L1-L6) and Dianshan lake (L7)).

Table S4. Vector loading of PBDEs congener in PC-1 and PC-2.

Table S5. Spearman’s rank correlation coefficient (r) for the correlation between selected PCB and PBDE congeners in Sediments.

Table S6. Biota-sediment accumulation factor (BSAF) of each PCBs and PBDEs congener.

Figure S1. Polychlorinated biphenyls (PCBs) congener profiles in snails ((*Bellamya Aeruginosa*, ♂: male; ♀: female) and sediments (S).

Figure S2. Polybrominated diphenyl ethers (PBDEs) congener profiles in snails ((*Bellamya Aeruginosa*, ♂: male; ♀: female) and sediments (S).

Figure S3. Principal component analysis (PCA), comparison of the congener profile of polybrominated diphenyl ethers (PBDEs) in snails (*Bellamya Aeruginosa*) and sediment to commercial technical mixtures of PBDEs.

Table S1. Detailed information and description of the sampling sites.

| Sampling site | Sampling area | Latitude and altitude | Sampling site description |
| --- | --- | --- | --- |
| L1 | East Taihu Lake | N 31˚01.178´  E 120˚29.671´ | Close to Suzhou city, grass type lake, relative good water quality, low dissolved oxygen (DO) content, outflow of the lake |
| L2 | Xiaomeikou | N 30˚56.292´  E 120˚07.831´ | Close to Huzhou city, cyanobacteria blooming place, inflow to the lake |
| L3 | Dapukou | N 31˚18.641´  E 119˚55.985´ | Close to Yixing area (Wuxi city), OCPs largely used for agriculture purpose, relatively poor water quality, main lake inflow |
| L4 | Zhushan Lake | N 31˚27.327´  E 120˚04.791´ | Close to Changzhou city, cyanobacteria blooming place, low DO content, overloading pollution area, inflow to the lake |
| L5 | Meiliang Bay | N 31˚26.734´  E 120˚12.610´ | Water sources of Wuxi city, urbanization area, cyanobacteria blooming place, inflow to the lake |
| L6 | Gong Lake | N 31˚26.734´  E 120˚12.610´ | Close to Wuxi city, rich in phytoplankton, high DO content, lake entrance |
| L7 | Dianshan lake | N 31˚08.055´  E 121˚00.579´ | In Shanghai, one of the drinking water sources for Shanghai, upstream of the Huangpu river. |

Table S2. Lipid content (%) and concentrations (ng g^-1^ lipid weight (lw)) of polychlorinated biphenyls (PCBs) and polybrominated diphenyl ethers (PBDEs) in snails (*Bellamya aeruginosa,* ♂: male and ♀: female) from the Taihu lake area (Taihu lake (L1-L6) and Dianshan lake (L7)).

|  | L1♂ | L1♀ | L2♂ | L2♀ | L3♂ | L3♀ | L4♂ | L4♀ | L5♂ | L5♀ | L6♂ | L6♀ | L7♂ | L7♀ |
| --- | --- | --- | --- | --- | --- | --- | --- | --- | --- | --- | --- | --- | --- | --- |
| Lipid content (%) | 2.0% | 1.8% | 1.4% | 1.4% | 1.6% | 1.5% | 1.6% | 1.8% | 1.5% | 1.5% | 1.6% | 2.1% | 1.5% | 1.5% |
| CB-28 | ND | ND | ND | ND | ND | ND | ND | ND | ND | ND | ND | ND | ND^a^ | ND |
| CB-52 | 4.0 | ND | ND | ND | ND | 22 | 82 | 75 | ND | ND | 19 | 15 | ND | ND |
| CB-101 | 17 | 12 | 5.0 | 5.0 | 54 | 35 | 71 | 62 | 7.1 | 9.3 | 22 | 21 | 21 | 8.8 |
| CB-118 | 22 | 15 | 8.3 | 8.1 | 31 | 20 | 78 | 68 | 12 | 16 | ND | 29 | 47 | 11 |
| CB-153 | 100 | 45 | 57 | 72 | 93 | 72 | 130 | 93 | 47 | 18 | 110 | 87 | 54 | 35 |
| CB-138 | 43 | 18 | 18 | 15 | 47 | 31 | 180 | 140 | 37 | 49 | 61 | 63 | 40 | 19 |
| CB-180 | 16 | ND | 11 | 11 | 45 | 29 | 49 | 43 | 8.8 | 12 | 23 | 18 | 11 | 5.6 |
| CB-194 | 3.9 | <LOQ^b^ | 3.5 | 3.3 | 5.0 | 2.9 | 3.9 | 4.8 | 4.0 | 3.6 | 4.7 | 4.2 | 2.8 | <LOQ |
| CB-195/208 | 2.2 | <LOQ | ND | ND | ND | ND | 5.2 | 3.7 | ND | ND | <LOQ | <LOQ | ND | <LOQ |
| CB-196/203 | 6.5 | 3.6 | 4.8 | 5.0 | 6.2 | 4.0 | 11 | 8.0 | 5.0 | 5.0 | 8.7 | 6.8 | 4.3 | 2.4 |
| CB-197 | <LOQ | <LOQ | <LOQ | <LOQ | ND | <LOQ | 3.7 | <LOQ | <LOQ | <LOQ | <LOQ | <LOQ | ND | ND |
| CB-198 | ND | ND | ND | ND | ND | ND | ND | ND | ND | ND | <LOQ | <LOQ | ND | ND |
| CB-199 | 3.0 | <LOQ | ND | ND | <LOQ | ND | 4.3 | 3.5 | 3.4 | <LOQ | <LOQ | 2.3 | ND | ND |
| CB-204/201 | <LOQ | <LOQ | 2.6 | <LOQ | 2.5 | <LOQ | 3.4 | <LOQ | ND | ND | <LOQ | <LOQ | ND | ND |
| CB-202 | ND | ND | ND | ND | ND | ND | ND | ND | ND | ND | ND | ND | ND | ND |
| CB-205 | <LOQ | ND | ND | ND | ND | ND | ND | ND | ND | ND | ND | ND | ND | ND |
| CB-206 | 4.4 | 2.3 | ND | ND | ND | ND | 7.9 | 5.7 | 6.2 | 5.5 | 5.1 | 4.2 | 3.6 | <LOQ |
| CB-207 | 3.1 | <LOQ | 3.5 | 3.2 | ND | ND | 6.9 | 4.9 | 7.4 | 5.9 | 4.1 | 3.4 | 2.7 | <LOQ |
| CB-209 | 6.1 | 2.1 | 4.6 | 5.6 | 5.7 | 4.0 | 40 | 20 | 18 | 14 | 17 | 9.8 | 7.8 | 3.0 |
| ∑7PCBs^a^ | 200 | 100 | 99 | 110 | 300 | 210 | 590 | 480 | 110 | 100 | 270 | 230 | 170 | 79 |
| ∑22PCBs^b^ | 240 | 120 | 120 | 130 | 320 | 220 | 680 | 540 | 160 | 140 | 320 | 270 | 190 | 90 |
|  |  |  |  |  |  |  |  |  |  |  |  |  |  |  |
| BDE-28 | <LOQ | <LOQ | <LOQ | <LOQ | 1.7 | <LOQ | 3.3 | 3.9 | ND | ND | 1.8 | <LOQ | 1.4 | <LOQ |
| BDE-47 | 10 | 6.6 | 12 | 13 | 17 | 14 | 21 | 24 | 5.1 | 7.0 | 22 | 14 | 9.8 | 4.9 |
| BDE-66 | 1.5 | 0.95 | 1.6 | 1.7 | ND | ND | 2.9 | 4.1 | 1.1 | 1.1 | 4.7 | 2.5 | 1.1 | <LOQ |
| BDE-99 | 3.9 | 4.0 | 11 | 10 | 22 | 14 | 20 | 20 | 11 | 8.5 | 17 | 11 | 13 | 7.2 |
| BDE-100 | 3.1 | 2.6 | 8.0 | 7.3 | 7.7 | 5.0 | 4.4 | 4.1 | 7.1 | 6.3 | 7.0 | 4.6 | 8.3 | 4.7 |
| BDE-153 | 2.2 | 1.9 | 13 | 16 | 9.5 | 7.0 | 12 | 15 | 4.3 | 5.1 | 9.8 | 9.8 | 4.0 | 2.4 |
| BDE-154 | 1.9 | 1.0 | 4.7 | 4.7 | 3.5 | 2.6 | 3.8 | 5.5 | ND | ND | 5.2 | 4.0 | 3.2 | 1.4 |
| BDE-183 | 1.9 | 1.2 | 7.5 | 6.5 | 4.8 | 3.1 | 8.2 | 6.3 | 2.9 | 2.9 | 4.6 | 5.0 | 3.1 | 1.1 |
| BDE-196 | <LOQ | ND | 5.6 | 4.4 | ND | 2.1 | 6.3 | 4.4 | ND | ND | 3.0 | 2.9 | ND | ND |
| BDE-197/04 | 9.2 | 4.1 | 12 | ND | ND | ND | 12 | 6.0 | 6.7 | 6.6 | 7.0 | 5.4 | 5.0 | ND |
| BDE-198/199  /200/203 | <LOQ | ND | 16 | 15 | 6.0 | 4.5 | 11 | 9.4 | 4.6 | 5.5 | 6.9 | 7.4 | 8.4 | 2.6 |
| BDE-201 | 4.2 | 2.2 | 11 | 9.3 | 6.1 | 3.5 | 14 | 8.8 | 5.7 | 4.6 | 9.6 | 9.5 | 6.1 | <LOQ |
| BDE-202 | 1.3 | ND | 4.1 | 3.7 | ND | ND | 5.5 | 4.6 | ND | ND | 3.6 | 4.6 | ND | ND |
| BDE-206 | ND | ND | 12 | 9.2 | ND | ND | 8.6 | 7.4 | ND | ND | ND | ND | ND | ND |
| BDE-207 | 4.0 | ND | 26 | 15 | 10 | 6.2 | 33 | 16 | 13 | 10 | 19 | 8.9 | 7.8 | ND |
| BDE-208 | ND | ND | 11 | 7.5 | ND | ND | 14 | 9.5 | ND | ND | 7.3 | 6.3 | ND | ND |
| BDE-209 | 9.5 | ND | 42 | 31 | ND | 21 | 57 | 27 | 19 | 19 | 38 | 18 | ND | ND |
| ∑_8_PBDEs^c^ | 25 | 19 | 59 | 61 | 66 | 47 | 76 | 84 | 3 | 31 | 72 | 51 | 44 | 23 |
| ∑_24_PBDEs^d^ | 56 | 25 | 200 | 170 | 89 | 84 | 240 | 180 | 80 | 77 | 170 | 110 | 71 | 27 |

1. ND: not detected, below limit of detection (LOD).
2. Below limit of quantification (LOQ) but above LOD. LOQ (ng g^-1^ lw) used in statistical calculations and based on average values and lipid weights:CB-194 (2.8), CB-195/208 (2.1), CB-196/203 (2.3), CB-197 (2.8), CB-198 (1.8), CB-199 (2.3), CB-204/201 (2.5), CB-205 (4.1), CB-206 (2.3), CB-207 (2.1); BDE-47 (1.3), BDE-66 (0.66), BDE-196 (2.0), BDE-198/199/200/203 (2.0), BDE-201 (2.0).
3. ∑_7_PCBs: sum of CB-28, -52, -101, -118, -138, -153 and -180.
4. ∑_22_PCBs sum of CB-28, -52, -101, -118, -138, -153, -180, -194, -195, -196, -197, -198, -199, -201, -202, -203, -204, -205, -206, -207, -208 and -209.
5. ∑_8_PBDEs: sum of BDE-28, -47, -66, -99, -100, -153, -154 and -183.
6. ∑_24_PBDEs: sum of BDE-28, -47, -66, -99, -100, -153, -154, -183, -194, -195, -196, -197, -198, -199, -200, -201, -202, -203, -204, -205, -206, -207, -208 and -209.

Table S3. Water content (%), total organic carbon content (%) and concentrations (ng g^-1^ dry weight (dw)) of polychlorinated biphenyls (PCBs) and polybrominated diphenyl ethers (PBDEs) in sediments from the Taihu lake area (Taihu lake (L1-L6) and Dianshan lake (L7)).

|  | L1 | L2 | L3 | L4 | L5 | L6 | L7 |
| --- | --- | --- | --- | --- | --- | --- | --- |
| water content (%) | 27 | 32 | 34 | 33 | 36 | 29 | 34 |
| Total organic carbon (%) | 0.60 | 0.68 | 0.94 | 1.2 | 1.1 | 0.78 | 1.5 |
| CB-28 | ND^a^ | ND | 0.086 | 0.096 | 0.039 | ND | NA^b^ |
| CB-52 | ND | ND | 0.072 | 0.15 | 0.039 | ND | NA |
| CB-101 | 0.026 | 0.010 | 0.049 | 0.076 | 0.022 | 0.051 | NA |
| CB-118 | 0.039 | 0.020 | ND | ND | 0.041 | 0.017 | NA |
| CB-153 | 0.055 | 0.049 | 0.18 | 0.18 | 0.048 | 0.045 | NA |
| CB-138 | 0.044 | 0.019 | 0.013 | 0.076 | 0.032 | 0.019 | NA |
| CB-180 | 0.010 | ND | ND | ND | 0.007 | ND | NA |
| CB-209 | 0.007 | 0.010 | 0.032 | 0.19 | 0.023 | 0.021 | 0.018 |
| ∑_7_PCBs^d^ | 0.17 | 0.097 | 0.40 | 0.57 | 0.23 | 0.13 | NA |
| ∑22PCBs^e^ | 0.019 | 0.11 | 0.43 | 0.82 | 0.25 | 0.15 | 0.018 |
|  |  |  |  |  |  |  |  |
| BDE-28 | <LOQ ^c^ | 0.005 | 0.008 | 0.009 | 0.004 | 0.004 | ND |
| BDE-47 | 0.009 | 0.028 | 0.023 | 0.033 | 0.016 | 0.017 | 0.010 |
| BDE-66 | <LOQ | 0.007 | ND | ND | 0.006 | ND | ND |
| BDE-100 | 0.020 | 0.024 | 0.028 | 0.031 | 0.014 | 0.023 | ND |
| BDE-99 | 0.040 | 0.042 | 0.065 | 0.087 | 0.030 | 0.046 | ND |
| BDE-154 | ND | 0.024 | 0.035 | 0.14 | 0.023 | 0.027 | 0.020 |
| BDE-153 | ND | 0.017 | 0.025 | 0.078 | 0.035 | 0.013 | ND |
| BDE-183 | <LOQ | 0.065 | 0.12 | 0.54 | 0.085 | 0.087 | 0.037 |
| BDE-194 | ND | ND | ND | ND | ND | ND | ND |
| BDE-195 | ND | ND | ND | ND | ND | ND | ND |
| BDE-196 | ND | 0.095 | 0.20 | 0.81 | 0.12 | 0.11 | 0.075 |
| BDE-197/204 | <LOQ | 0.066 | 0.13 | 0.63 | 0.10 | 0.10 | 0.083 |
| BDE- 198/199/200/203 | <LOQ | 0.11 | 0.22 | 0.87 | 0.14 | 0.12 | 0.029 |
| BDE-201 | <LOQ | 0.16 | 0.30 | 1.5 | 0.23 | 0.25 | 0.11 |
| BDE-202 | ND | 0.058 | 0.11 | 0.54 | 0.078 | 0.095 | 0.030 |
| BDE-205 | ND | ND | ND | ND | ND | ND | ND |
| BDE-206 | 0.021 | 0.20 | 0.48 | 2.0 | 0.26 | 0.24 | 0.30 |
| BDE-207 | <LOQ | 0.25 | 0.47 | 2.1 | 0.31 | 0.30 | 0.66 |
| BDE-208 | <LOQ | 0.16 | 0.33 | 1.4 | 0.22 | 0.22 | 0.38 |
| BDE-209 | 0.45 | 5.5 | 7.0 | 56 | 4.8 | 4.6 | 5.6 |
| ∑_8_PBDEs^f^ | 0.078 | 0.21 | 0.30 | 0.92 | 0.21 | 0.22 | 0.067 |
| ∑_24_PBDEs^g^ | 0.63 | 6.8 | 9.6 | 67 | 6.5 | 6.3 | 7.7 |

1. ND: not detected, below limit of detection (LOD).
2. NA: not analyzed.
3. Below limit of quantification (LOQ) but above LOD. LOQ (ng g^-1^ dw) used in statistical calculations and based on average values and dry weight: BDE-28 (0.0038), BDE-66 (0.0038), BDE-183 (0.015), BDE-197/204 (0.017), BDE-198/199/200/203 (0.023), BDE-201 (0.028), BDE-207 (0.055) and BDE-208 (0.090).
4. ∑_7_PCBs: sum of CB-28, -52, -101, -118, -138, -153 and -180.
5. ∑_22_PCBs sum of CB-28, -52, -101, -118, -138, -153, -180, -194, -195, -196, -197, -198, -199, -201, -202, -203, -204, -205, -206, -207, -208 and -209.
6. ∑_8_PBDEs: sum of BDE-28, -47, -66, -99, -100, -153, -154 and -183.
7. ∑_24_PBDEs: sum of BDE-28, -47, -66, -99, -100, -153, -154, -183, -194, -195, -196, -197, -198, -199, -200, -201, -202, -203, -204, -205, -206, -207, -208 and -209.

Table S4. Vector loading of PBDEs congener in PC-1 and PC-2.

|  | PC-1 | PC-2 |
| --- | --- | --- |
| BDE 28 | 0,00 | 0,00 |
| BDE 47 | -0,30 | -0,43 |
| BDE 66 | 0,00 | 0,00 |
| BDE 100 | -0,08 | -0,11 |
| BDE 99 | -0,34 | -0,50 |
| BDE 154 | -0,03 | -0,02 |
| BDE 153 | -0,06 | 0,05 |
| BDE 183 | -0,07 | 0,56 |
| BDE 201 | 0,00 | 0,01 |
| BDE 197 | -0,03 | 0,31 |
| BDE 198,199,200,203 | 0,00 | 0,09 |
| BDE 196 | -0,02 | 0,14 |
| BDE 208 | 0,00 | 0,00 |
| BDE 207 | 0,01 | 0,17 |
| BDE 206 | 0,04 | 0,03 |
| BDE 209 | 0,88 | -0,29 |

Table S5. Spearman’s rank correlation coefficients (r) for the correlation between selected PCB and PBDE congeners in Sediments.

|  |  | BDE-47 | BDE-100 | BDE-99 | BDE-153 | BDE-183 | BDE-207 | BDE-209 | CB-209 | CB-101 | CB-153 | CB-138 |
| --- | --- | --- | --- | --- | --- | --- | --- | --- | --- | --- | --- | --- |
| BDE-47 | correlation coefficient （r） |  |  |  |  |  |  |  |  |  |  |  |
| BDE-100 | correlation coefficient （r） | 0.89* |  |  |  |  |  |  |  |  |  |  |
| BDE-99 | correlation coefficient （r） | 0.77 | 0.94** |  |  |  |  |  |  |  |  |  |
| BDE-153 | correlation coefficient （r） | 0.60 | 0.43 | 0.37 |  |  |  |  |  |  |  |  |
| BDE-183 | correlation coefficient （r） | 0.66 | 0.71 | 0.83* | 0.72 |  |  |  |  |  |  |  |
| BDE-207 | correlation coefficient （r） | 0.60 | 0.60 | 0.66 | 0.89* | 0.94** |  |  |  |  |  |  |
| BDE-209 | correlation coefficient （r） | 0.89* | 0.83* | 0.71 | 0.83* | 0.77 | 0.83* |  |  |  |  |  |
| CB-209 | correlation coefficient （r） | 0.60 | 0.60 | 0.66 | 0.89* | 0.94** | 1.0** | 0.83* |  |  |  |  |
| CB-101 | correlation coefficient （r） | 0.31 | 0.54 | 0.77 | 0.26 | 0.77 | 0.60 | 0.31 | 0.60 |  |  |  |
| CB-153 | correlation coefficient （r） | 0.37 | 0.66 | 0.54 | 0.31 | 0.37 | 0.43 | 0.60 | 0.43 | 0.26 |  |  |
| CB-138 | correlation coefficient （r） | -0.086 | -0.086 | -0.029 | 0.20 | 0.029 | 0.086 | -0.086 | 0.086 | 0.37 | -0.029 |  |

*. Significant at α=0.05 level (double sided); **. Significant at α=0.01 level (two tailed)

Table S6. Biota-sediment accumulation factor (BSAF) of each PCBs and PBDEs congener

| Congener | Number of bromine | BSAF | Congener | Number of chlorine | BSAF |
| --- | --- | --- | --- | --- | --- |
| BDE-28 | 3 | 2.3 | CB-52 | 4 | 4.8 |
| BDE-47 | 4 | 6.5 | CB-101 | 5 | 5.5 |
| BDE-66 | 4 | 2.2 | CB-118 | 5 | 5.6 |
| BDE-99 | 5 | 2.2 | CB-153 | 6 | 8.8 |
| BDE-100 | 5 | 2.3 | CB-199 | 8 | 5.4 |
| BDE-153 | 6 | 3.7 | CB-206 | 9 | 5.8 |
| BDE-154 | 6 | 0.92 | CB-209 | 10 | 3.8 |
| BDE-183 | 7 | 0.66 |  |  |  |
| BDE-196 | 8 | 0.11 |  |  |  |
| BDE-197 | 8 | 1.1 |  |  |  |
| BDE-198/199/200/203 | 8 | 0.79 |  |  |  |
| BDE-201 | 8 | 0.43 |  |  |  |
| BDE-202 | 8 | 0.15 |  |  |  |
| BDE-206 | 9 | 0.06 |  |  |  |
| BDE-207 | 9 | 0.31 |  |  |  |
| BDE-208 | 9 | 0.11 |  |  |  |
| BDE-209 | 10 | 0.031 |  |  |  |

Figure S1. Polychlorinated biphenyls (PCBs) congener profiles in snails ((*Bellamya Aeruginosa*, ♂: male; ♀: female) and sediments (S).

Figure S2. Polybrominated diphenyl ethers (PBDEs) congener profiles in snails (*Bellamya Aeruginosa*, ♂: male; ♀: female) and sediments (S).
